# 2D Semiconductor Nanosheets Supported on Colloidal Quantum Cubes

**DOI:** 10.1021/acsnano.6c03530

**Published:** 2026-06-22

**Authors:** Divesh Nazar, Dulanjan Harankahage, Annelies Vitharana, Benjamin T. Diroll, Mykhailo V. Bondarchuk, Christopher M. Hicks, Sean Smith, Dmitry Porotnikov, Amelia D. Waters, Siddhartha Thennakoon, Kosgoda Somarathne, Joelle DesAutels, Issatay Nadinov, Krishna P. Acharya, Richard D. Schaller, Alexander N. Tarnovsky, Anton V. Malko, Pieter Geiregat, Mikhail Zamkov

**Affiliations:** † The Center for Photochemical Sciences, 1888 Bowling Green State University, Bowling Green, Ohio 43403, United States; ‡ Department of Physics, Bowling Green State University, Bowling Green, Ohio 43403, United States; § Physics and Chemistry of Nanostructures, 26656 Ghent University, 9000 Gent, Belgium; ∥ NOLIMITS Center for Non-Linear Microscopy and Spectroscopy, Ghent University, 9000 Gent, Belgium; ⊥ 1291 Center for Nanomaterials, Argonne National Laboratory, Lemont, Illinois 60439, United States; # Department of Chemistry, Bowling Green State University, Bowling Green, Ohio 43403, United States; ∇ Department of Physics, 12335 The University of Texas at Dallas, Richardson, Texas 75080, United States; ○ Center for Renewable Energy and Storage Technologies (CREST), Division of Physical Sciences and Engineering, 127355 King Abdullah University of Science and Technology (KAUST), Thuwal 6900, Kingdom of Saudi Arabia; ◆ 1073 Savannah River National Laboratory, Aiken, South Carolina 29808, United States; ¶ Department of Chemistry, Northwestern University, Evanston, Illinois 60208, United States

**Keywords:** nanoplatelets, entanglement, colloidal quantum wells, semiconductor nanocrystals, stimulated emission

## Abstract

Two-dimensional (2D) colloidal nanocrystals bridge the physics of epitaxial quantum wells with the synthetic scalability of solution-processed nanomaterials, making them an attractive platform for future LEDs, lasers, and photodetectors. However, their strongly anisotropic 2D morphology complicates the formation of close-packed films required for electrically interfaced devices. Here, we address this limitation by introducing semiconductor quantum cubes (QCs) in which 2D CdSe nanosheets are conformally grown on six faces of CdS cubic scaffolds. This architecture preserves excitonic physics of quasi-2D nanosheets while leveraging the mechanical rigidity and self-assembly of nanocubes, enabling close-packed films with improved electronic coupling and electrical conductivity. By extending CdS/CdSe QCs into a CdS/CdSe/CdS core/shell/shell structure, we realize 2D quantum wells that exhibit unusual multiexciton behavior. In particular, CdS/CdSe/CdS QCs show repulsive exciton–exciton interactions, which decouple CdSe facets into quasi-independent emitters. This leads to the suppressed Auger recombination, long multiexciton lifetimes, and broadband optical gain. The demonstrated combination of unusual multiexciton photophysics and favorable film self-assembly characteristics in QCs presents opportunities for solution-processed optoelectronic devices.

## Graphical abstract

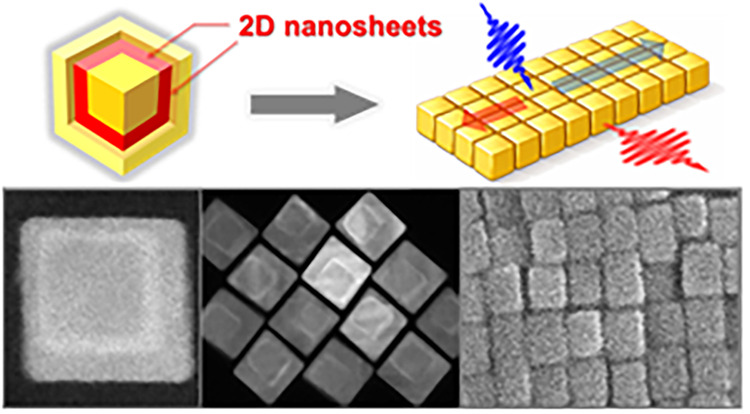

## Introduction

Two-dimensional (2D) colloidal semiconductor nanocrystals (NCs) resemble the electronic structure of semiconductor quantum wells while offering a synthetic scalability of colloidal nanomaterials.^1–10^ In 2D NCs, such as nanoplatelets (NPLs),^1^ nanosheets,^3^ and nanoribbons,^2^ strong out-of-plane quantum confinement gives rise to large exciton binding energies,^11^ giant oscillator strengths,^12^ and exceptionally narrow emission line widths.^13,14^ These properties have positioned 2D colloidal semiconductors as promising materials for optoelectronic technologies, particularly in low-threshold optical gain media^15–17^ and high efficiency, color-pure light-emitting diodes (LEDs).^18,19^ Furthermore, the coherent and directional emission from 2D NCs^20^ makes them promising candidates for emerging optoelectronic phenomena, such as surface-spin polarized exciton emission,^21^ electron shakeup,^22^ and quantum light generation.^23–25^

Despite their promise in prototype optoelectronic devices,^26,27^ 2D colloidal NCs still face barriers to practical implementation in scalable photonic platforms. Their sheet-like morphology can make them mechanically fragile^28,29^ and difficult to assemble into electrically coupled films on substrates,^30–35^ unless specialized deposition techniques are applied.^36,37^ For instance, films derived from 2D NPLs using simplified deposition techniques, such as spin coating or drop casting, often exhibit weak interparticle coupling^38^ with a substantial positional and orientational disorder,^36^ which hinders charge transport and suppresses coherent electronic coupling. As a result, long-range behavior, including miniband transport,^39^ sequential resonant tunneling,^40^ or quantum-cascade-like architectures,^6^ could be difficult to scale for large-area substrates. Additional challenges arise from the very confinement effects that sharpens emission in 2D NCs, as these can also accelerate exciton and biexciton recombination, thereby increasing optical gain thresholds under device-relevant excitation conditions.^41^ Together, these limitations justify the need for additional improvements in present 2D NC geometries.

Here, we introduce a quantum cube (QC) architecture in which 2D CdSe excitonic layers are grown on all six faces of 3D CdS colloidal nanocube scaffolds (Figure 1a). In this geometry, the CdS cube provides a rigid three-dimensional support that overcomes several limitations of freestanding 2D NCs. In particular, QC films prepared by simple drop casting, form more ordered, densely packed assemblies with improved electrical conductivity compared to conventional 2D NC films. At the same time, the CdSe facets of QCs preserve the essential excitonic characteristics of 2D quantum wells, which are further modified by an unusual regime of exciton–exciton repulsion. This repulsive interaction suppresses Auger recombination, giving rise to long-lived, broadband optical gain with each CdSe facet acting as a quasi-independent emitter.

**Figure 1:**
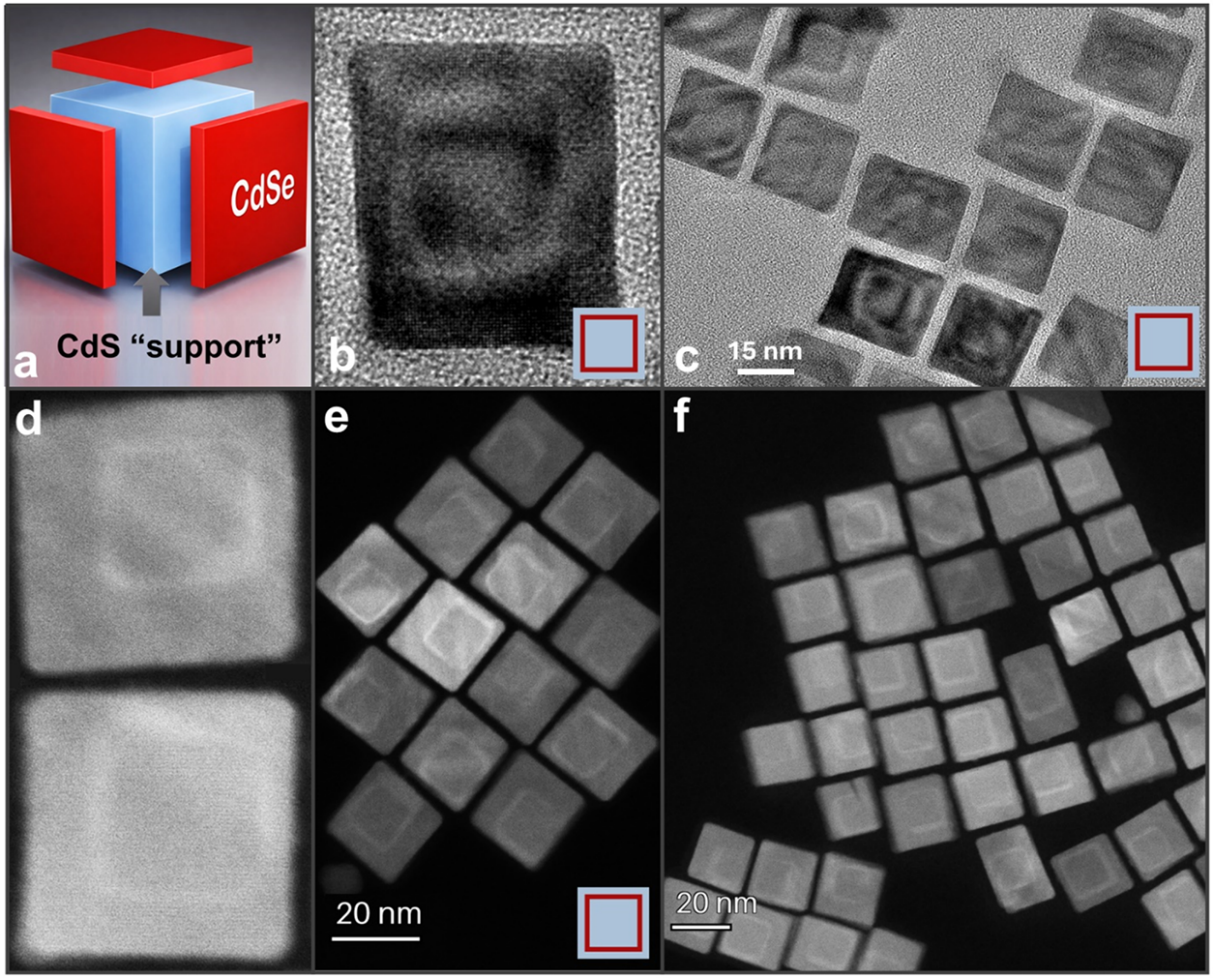
(a) Schematics of a QC morphology comprising six 2D CdSe nanosheets conformally supported on the faces of a CdS colloidal nanocube. (b, c) TEM images of CdS/CdSe/CdS core/shell/shell QCs. (d–f) High-angle annular dark field scanning transmission electron microscopy (HAADF-STEM) images of QCs showing the location of the CdSe 2D nanosheets as a lighter contrast relative to the CdS core.

## Results and Discussion

The QC structure consists of a three-dimensional CdS cubic core that supports 2D CdSe shell layers on its six faces (Figure 1a). Cubic CdS/CdSe core/shell NCs can be further overcoated with an outer shell of CdS to improve surface passivation, thus yielding an overall CdS/CdSe/CdS core/shell/shell QC. Since the edge length of the CdS core (5–10 nm) exceeds the CdS exciton Bohr radius (∼3.4 nm), the CdS cube behaves essentially as a bulk-like domain, with quantum confinement existing primarily in the CdSe shell. TEM images in Figure 1b,c confirm the overall cubic morphology of CdS/CdSe/CdS QCs, while HAADF-STEM images in Figure 1d–f reveal the CdSe nanosheets as regions of lighter contrast surrounding the CdS core, consistent with conformal 2D CdSe coverage on each face.

The CdS core NCs were synthesized using an aggregative growth method adapted from previous protocols.^42–44^ This process employs small, 2.5–3.0 nm, zinc blende CdS NCs that assemble into larger cubes through fusion. The fusion processes are initiated by the addition of stearoyl chloride either with or without a supplemental sulfur precursor, which drives the aggregation of original NCs followed by their thermodynamic reconstruction into monodisperse, single-crystalline cubes with well-defined {100} faceting (Figure S1). The kinetics of this fusion-type growth are revealed by tracking the evolution of the photoluminescence (PL) peak. For this purpose, we use brighter-emitting CdSe NCs, whose PL features are more easily resolved, and initiate fusion growth by adding stearoyl chloride. As shown in Figure S4, the original NCs evolve toward a larger thermodynamically favored size, reflected by a stepwise red shift of the PL peak that is consistent with discrete increases in cube volume. At intermediate temperatures, the PL spectra become bimodal, with the original small-size particle emission coexisting alongside a new red-shifted band arising from the coalesced structures. At the final growth temperature, the reaction mixture is allowed to size focus for 3–5 min, ultimately resulting in a narrow PL line width. For the CdS cubes, the final PL maximum is centered near 512 nm (Figure 2c), approaching its respective bulk value. The use of aforementioned thermodynamic growth conditions was essential for realizing monocrystalline cubic supports capable of accommodating 2D excitonic layers via heteroepitaxy. The reaction temperature served as the primary parameter for tuning the cube edge length. High-resolution TEM (Figure S1e) confirms a single-phase zinc-blende crystal structure of CdS core cubes.

**Figure 2:**
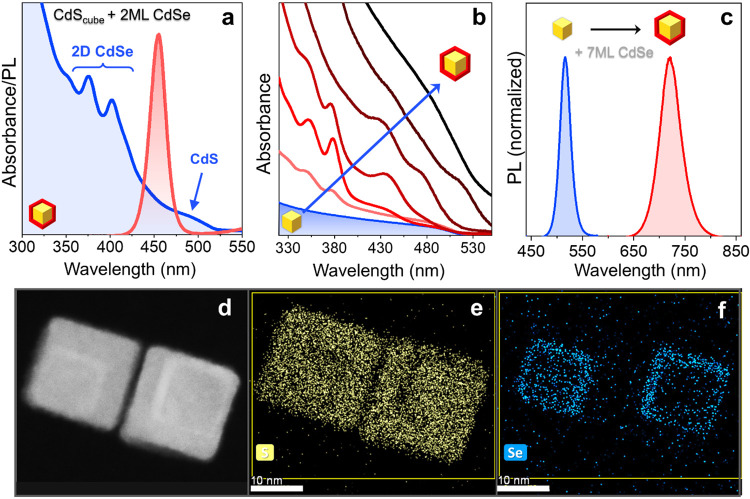
(a) Absorption and emission spectra of CdS/CdSe QCs featuring ∼2-monolayer CdSe nanosheets grown on CdS nanocube facets. Distinct CdSe absorption features appear at ∼375 nm and ∼400 nm, consistent with light-hole and heavy-hole like transitions, respectively. (b) Evolution of the CdS/CdSe QC absorption during the CdSe shell growth, highlighting the gradual change in the CdSe quantum well width. (c) PL spectra of CdS cubes and CdS/CdSe core/shell QCs. (d) HAADF-STEM image of CdS/CdSe/CdS QCs, showing the morphology of the CdSe nanosheet layer as a lighter contrast. (e, f) EDX elemental maps of the QCs shown in (d), indicating the spatial distribution of sulfur (e, yellow) and selenium (f, blue) within the CdS/CdSe/CdS heterostructure.

Deposition of 2D CdSe shell layers on CdS cubes was performed by a slow injection of the selenium precursor using a syringe pump with the cadmium precursor already present in the flask. This approach maintains a low instantaneous monomer concentration and promotes epitaxial CdSe growth across the {100} facets. As the CdSe layer first nucleates on cube faces, the absorption spectrum develops nanoplatelet like excitonic signatures (Figure 2a), with heavy and light hole transitions at energies consistent with an effective ∼2-monolayer CdSe thickness (Figure S3a). With continued CdSe deposition, nanosheets thicken and the initially sharp 2D excitonic features progressively broaden (Figures 2b, and S3a), similar to the evolution observed in conventional CdSe NPLs upon shell growth.^18^ At early stages, the CdS/CdSe heterostructure exhibits a type-II carrier localization regime, evidenced by a red-shifted, low intensity PL (Figures 2a and S3b) reflecting a reduced electron–hole overlap across the CdS/CdSe interface.

Following the CdSe layer deposition, CdS/CdSe core/shell NCs were further overcoated with a wider-bandgap CdS shell to create a barrier to surface charges. The absorption and emission spectra of the resulting CdS/CdSe/CdS QCs are shown in Figure S2d, with the detailed spectral evolution for the CdS/CdSe → CdS/CdSe/CdS step, presented in Figure S3c,d. HAADF-STEM images of the final QC structure (Figure 1d–f) reveal conformal CdSe layers, visible as lighter-contrast regions, which are sandwiched between the cubic CdS core and the outer CdS shell domains. Corresponding HAADF-STEM energy-dispersive X-ray (EDX) maps in Figures 2d–f and S3f confirm that selenium remains confined to the shell regions with minimal penetration into the CdS core. Analysis of the high-resolution images in Figure S2 shows that, for QCs emitting near λ = 700 nm, the average CdSe layer thickness is approximately 1.6 nm (about 5–6 monolayers (MLs)). We note that 5-ML CdSe NPLs capped with 7-ML CdS shells exhibit emission in a similar 650–700 nm range.^45,46^ Notably, QC samples emitting near 680 nm display the highest PL quantum yields (∼50–60%), suggesting an optimal balance between sufficient CdSe thickness to enhance oscillator strength and effective carrier confinement by the outer CdS shell.

The PL lifetime of a representative QC sample (Figure 3b, red curve) was found to be ∼25 ns. This value is substantially longer than the 4–6 ns lifetimes typically reported for 2D CdSe or CdSe/CdS core/crown NPLs,^47–49^ as well as the ∼10 ns lifetimes observed in isotropic-shell CdSe/CdS NPLs.^50^ The slower decay in QCs can be attributed to enhanced out-of-plane carrier delocalization of charges into the adjacent CdS core and shell domains. Consistent with the band alignment between zinc blende CdSe and CdS (Figure S5),^51^ photogenerated holes remain confined within the narrower gap CdSe facets, while electrons can delocalize into the larger volume of the CdS cubic core and surrounding shell regions. The increased spatial separation reduces electron–hole wave function overlap and suppresses radiative recombination, resulting in the longer PL lifetime.

**Figure 3:**
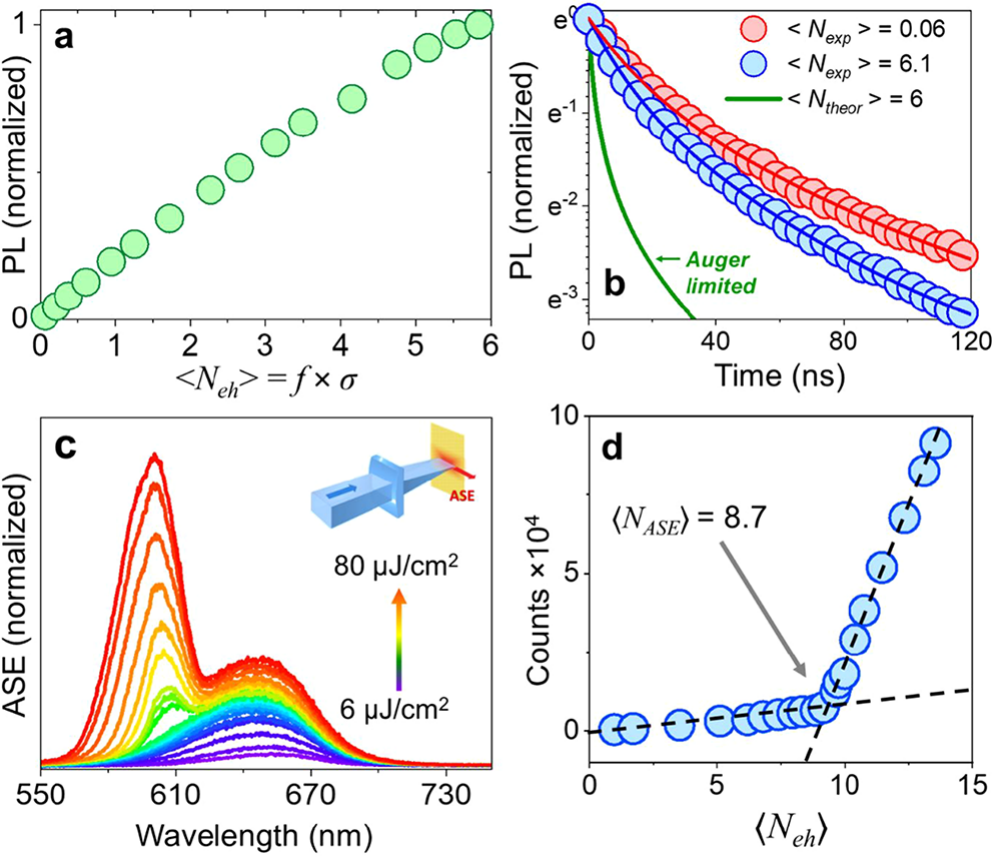
(a) Power dependent integrated PL intensity of QCs plotted as a function of the average number of absorbed photons per particle, ⟨*N*_eh_⟩. The PL intensity remains almost linear up to ⟨*N*⟩ ≈ 5–6, indicating efficient radiative recombination in the multiexciton regime. (b) Time-resolved PL decay traces measured at low excitation power (⟨*N*_eh_⟩ = 0.06) and high excitation power (⟨*N*_eh_⟩ = 6.1) conditions on a semi log scale. The high excitation PL decay shows an accelerated decay rate reflecting the increased contribution of fast decaying multiexciton states; however, this increase is significantly slower than expected theoretically for ⟨*N*_eh_⟩ = 6 (green curve). (c) Power dependent PL spectra from a QC film under stripe excitation (cylindrical lens), revealing the buildup of amplified spontaneous emission (ASE) on the high energy side of the single exciton PL band, consistent with ASE originating from two (or more) repulsive excitons. (d) Integrated ASE intensity from (c) as a function of ⟨*N*_eh_⟩, showing a clear nonlinear onset of amplification at ⟨*N*_eh_⟩ ≈ 8.7.

Further insights into the exciton recombination were obtained from the excitation power dependence of the PL intensity (Figure 3a) and PL lifetime (Figure 3b, blue curve). By using a sufficiently high pump fluence, we reach a regime for which the average number of photogenerated electron–hole pairs per particle, ⟨*N*_eh_⟩, exceeded unity, corresponding to multiexciton occupancy within a single cube. In conventional 0D QDs, creating two excitons almost always corresponds to forming a biexciton, which carries a high probability of nonradiative Auger recombination. This trend changes in 2D NCs, where biexcitons can coexist with noninteracting excitons and free carriers, making the PL power dependence more complex.^52,53^ QCs may exhibit a similar regime. Regardless of whether the excited-state population is better described as correlated multiexcitons or a partially ionized free-carrier ensemble, the nonradiative Auger rate is expected to increase more rapidly with carrier density than the radiative rate at room temperature. This imbalance reduces the multiexciton PL quantum yield (QY) and drives PL saturation for ⟨*N*_eh_⟩ > 1, a behavior commonly modeled using a 1 – exp­(−*N*_eh_⟩) dependence. The actual slope of the PL intensity versus ⟨*N*_eh_⟩ is simply the multiexciton PL QY (⟨*N*_eh_⟩), which drops sharply for ⟨*N*_eh_⟩ > 1 at room temperature.^54–56^

According to Figure 3a, PL quenching in QCs occurs at a relatively large ⟨*N*_eh_⟩. The PL intensity remains nearly linear with ⟨*N*_eh_⟩, and begins to saturate only when ⟨*N*_*eh*_⟩ approaches ∼6, consistent with strong suppression of multicarrier nonradiative decay. This interpretation is further supported by the PL intensity decay measurements in Figure 3b (blue curve). At a high pump fluence, corresponding to ⟨*N*_eh_⟩ = 6.1, the PL decay is expected to accelerate significantly due to increasing contribution from nonradiative recombination of multiexciton states^57^ (Figure 3b, green curve), however, the measured PL decay rate is only slightly faster than that observed under low-fluence excitation (⟨*N*_eh_⟩ = 0.06, red curve). This behavior suggests that either the nonradiative Auger decay from states with up to 6 electron–hole pairs is strongly suppressed or that multiple excitons are largely noninteracting. The latter scenario could potentially be rationalized by the fact that individual excitons may reside on different CdSe facets within each quantum cube thereby weakening the Coulomb coupling between initial biexcitonic and final excitonic states.^58^ The spatial separation of multiple excitons across different facets is consistent with the mutual repulsion of multiple holes confined within CdSe regions of a cube, without being sufficiently screened by delocalized electrons.

To further investigate the nature of exciton–exciton interactions in QCs, we have studied the onset of amplified spontaneous emission (ASE) from QC films. To this end, femtosecond laser pulses were shaped into a stripe with a cylindrical lens, allowing ASE to build up along the excitation path and be collected from the film edge. As shown in Figure 3c, increasing pump fluence produces a narrow ASE band centered at λ ≈ 601 nm, which is blue-shifted relative to the original PL maximum. This blue shift corresponds to a positive biexciton binding energy of Δ_XX_ = +140 meV and provides direct evidence of exciton–exciton repulsion in QCs. While repulsive interactions have been previously observed in type-II quantum dots, CdSe/CdTe core/crown NPLs,^59^ and quantum shells,^60–70^ they are uncommon in conventional 2D NPLs, where attractive interactions typically lead to red-shifted gain.^52,71,72^ The observed exciton repulsion in QCs is consistent with a partial spatial separation of excitons across different CdSe facets. Notably, the ASE threshold occurs at ⟨*N*_eh_⟩ ∼ 9 excitons per particle (Figure 3d), which is higher than the lowest thresholds reported for standalone 2D nanocrystals of ⟨*N*_eh_⟩ < 1.^73–75^ This difference can be understood in terms of exciton–exciton repulsion within a cube, which causes individual CdSe facets to emit in a quasi-independent manner. Namely, at the ASE threshold of ⟨*N*_eh_⟩ ∼ 9, excitons are expected to be distributed across the six facets, reducing the effective occupancy to about 9/6 = 1.5 excitons per nanosheet, a value comparable to ASE thresholds in standalone NPLs. We also note that the threshold may be further increased by additional optical losses, such as light scattering in the somewhat “oily” QC films. To test whether the different CdSe facets within a cube indeed behave as quasi-independent emitters, such that population inversion must be established on individual CdSe sheets, we next turn to transient absorption and single-particle PL measurements.

Single-particle PL measurements provide a direct probe of how individual CdSe facets within a QC contribute to emission. At a relatively low excitation fluences (⟨*N*⟩ < 0.5), we observed two distinct populations of QCs. A smaller fraction of QCs exhibited strong PL intermittency with emission remaining mostly in the off state, consistent with the presence of surface charging (Figure S6a). The second population (majority of QCs) exhibited suppressed blinking (Figure S6b), but with several quasi-stable PL intensity levels rather than the binary on/off blinking typical of single emitters (Figures 4a and S6c). Such behavior is often attributed to charged exciton states and usually appears at high excitation fluences; however, our measurements were performed at low excitation densities ruling out this mechanism. Importantly, time-resolved PL decays acquired while the emitter resides in different intensity plateaus (Figure 4c) yield nearly identical PL lifetimes of about 10–12 ns within experimental uncertainty. This behavior is inconsistent with trions or other charged-exciton processes, which typically exhibit shorter decay times. We therefore assign the plateaus to stochastic activation of independent emissive facets, associated with six weakly coupled CdSe nanosheets residing on the faces of a CdS cube. This interpretation is further supported by second-order (*g* ^(2)^(τ)) photon autocorrelation measurements, Figure 4d,e. Here, the value of the normalized correlation peak at delay time τ = 0 is a measure of coincidence nature of the emitted photon pairs. As has been shown before,^65,76^ the nonzero value is indicative of either a nonzero biexciton quantum yield or the presence of multiple independent quantum emitters. To separate the two mechanisms, we performed time-gated correlation measurements. At zero gate delay, the corresponding value of *g* ^(2)^(τ = 0) is 0.94 (Figure 4d), however, at the gate delay of ∼50 ns (Figure 4e), the value of the middle peak is still above 0.5. If the τ = 0 signal was dominated by biexciton emission, time gating would strongly suppress the central peak, instead, the observed trend is more consistent with the presence of multiple emitters. Together, these results are consistent with the presence of several quasi-independent emissive centers associated with distinct cube facets.

**Figure 4:**
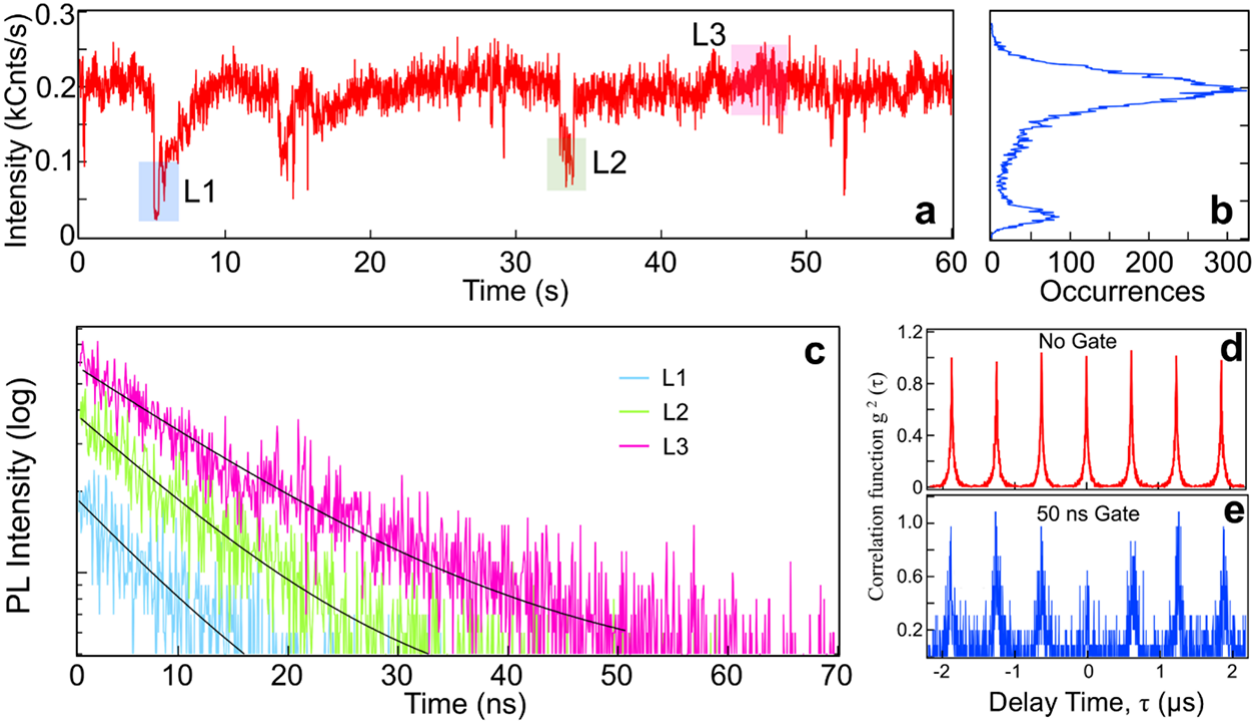
(a) Representative single QC PL trajectory showing several discrete, quasi-stable intensity plateaus (b) Histogram of blinking occurrences. (c) Time-resolved PL decays recorded while the emitter resides in different intensity plateaus, color-coded to the levels in panel (a). (d) Second-order photon cross-correlation when no gate to separate exciton and biexciton photons is applied. (e) Same data stream as used for panel (d), but now using a time gate of 50 ns to separate exciton and biexciton photons.

Further insights into multiexciton dynamics of QCs were revealed by femtosecond transient absorption (TA) spectroscopy, performed using λ = 375 nm pump pulses, and the probe window covering both the CdSe (570–710 nm) and the CdS core (<520 nm) absorbing transitions, as illustrated in Figure 5a. A representative TA spectrum is shown in Figure 5b, displaying a series of bleach spectra (Δ*A* < 0) resulting from photoexcitation at an average occupation of ⟨*N*_eh_⟩ ≈ 1 e–h pairs per QC (absorption cross section σ_3.1eV_ = 2.7 × 10^–13^ cm^2^; see Section S2). Two distinct bleach bands appear at λ ∼ 500 nm and λ ∼ 640 nm and were assigned to state filling in the CdS and the CdSe domains, respectively. Time-dependent analysis of the spectrally integrated TA signals (Figure S7) shows a progressive increase in the CdSe to CdS bleach ratio, revealing carrier redistribution after photoexcitation and indicating an efficient charge transfer from CdS to the CdSe quantum-well facets. This behavior is consistent with a quasi-type-II band alignment at the CdS/CdSe interface, in which photogenerated holes localize in the CdSe layer while electrons remain largely delocalized across the CdS scaffold.

**Figure 5:**
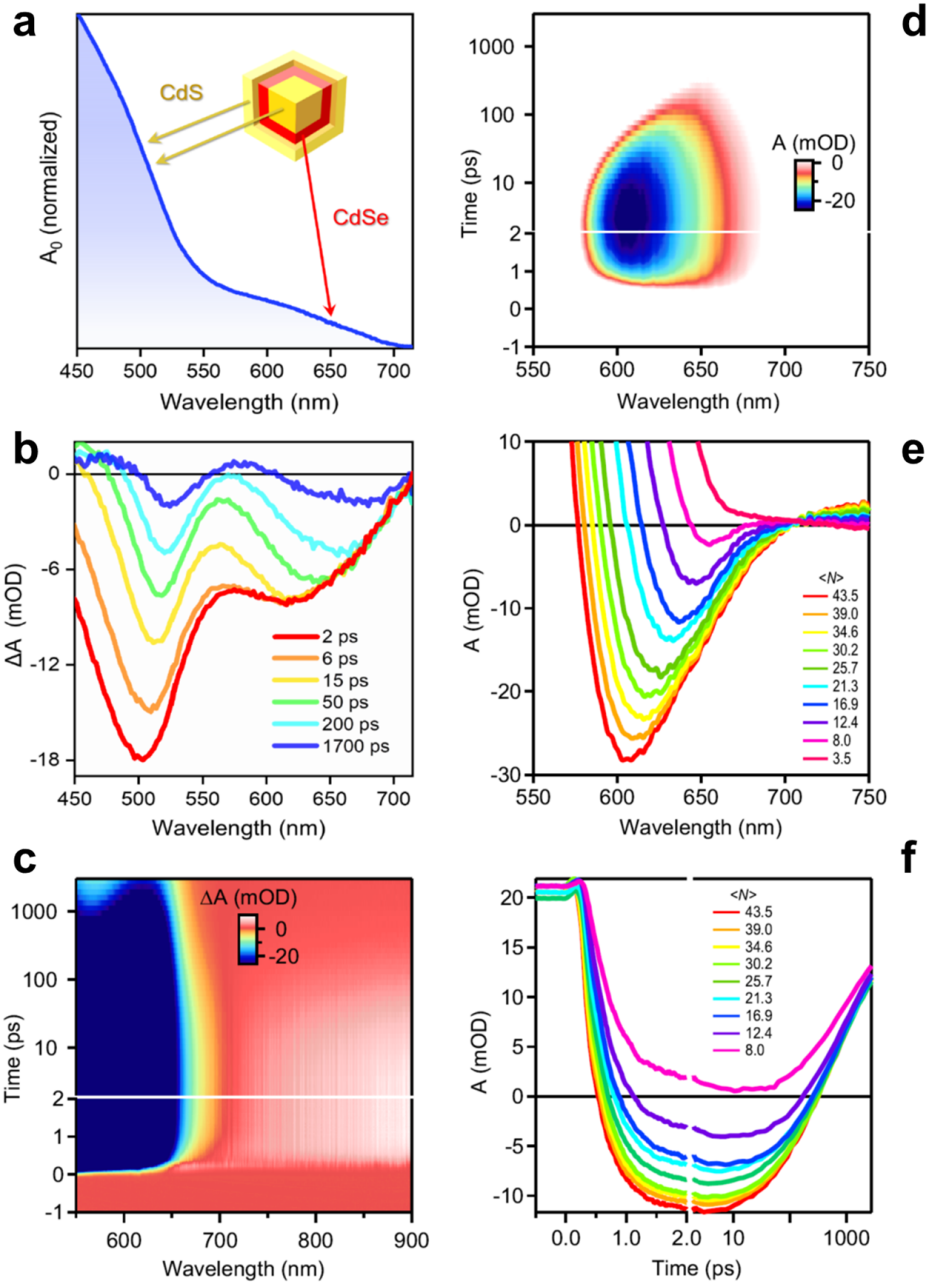
(a) Steady-state absorption spectrum of QCs with ∼1.6 nm CdSe quantum wells, highlighting contributions from both the CdS regions and the quantum-confined CdSe facets. (b) Femtosecond TA spectrum recorded at an average excitation density of ⟨*N*⟩ ≈ 1 using a 375 nm pump, showing a broad bleach across the 520–720 nm spectral window, assigned to state filling in the CdSe shell and a 450–500 nm bleach attributed to CdS core and shell regions. (c) False color map of ΔA resulting from the sample excitation at 515 nm, which shows both bleach (Δ*A* < 0) and photoinduced absorption (PA, Δ*A* > 0) regions. (d) Spectral and temporal dependence of the optical gain (Δ*A* + *A* < 0) measured at the highest pump fluence. (e) The gain spectra corresponding to the average occupation, ⟨*N*_eh_⟩, showing increasing gain amplitude and progressive broadening toward higher photon energies. (f) Time evolution of the optical gain measured at 650 nm, revealing the longest gain lifetimes of 330–340 ps at high fluences.

To avoid early time carrier thermalization and the charge transfer regime associated with pump pulses, we proceed to excite QCs at 515 nm, for which a false color map of ΔA < 0 is shown in Figure 5c. These measurements extend the probe into the longer wavelengths (>700 nm), where a distinct photoinduced absorption (PA, Δ*A* > 0) is seen in addition to a negative TA signal (Figure S8c). Upon increasing the excitation fluence, multiexciton populations start to dominate, leading to a regime where the net absorbance becomes negative (Δ*A* + *A*_0_ < 0). This indicates the onset of optical gain as the stimulated emission exceeds absorption. The wavelength and time dependence of this gain window at the highest fluence is summarized through a false color map in Figure 5d, highlighting the spectral-temporal regions where *A* = Δ*A* + *A*_0_ < 0. The corresponding fluence dependence of the gain response is shown in Figure 5e and the time evolution of the spectrally integrated gain is plotted in Figure 5f.

Initially, a positive photoinduced absorption partially offsets the developing net gain, but clear signatures of stimulated emission, Δ*A* + *A*_0_ < 0, are still evident (Figure S8a). After subtracting the PA contribution (Figure 5e), the gain threshold is observed at ⟨*N*_eh_⟩ ≈ 6.2, as confirmed by a more detailed fit in Figure S8b. At low excitation fluences (Figure 5e, ⟨*N*_eh_⟩ ≈ 8), a narrow gain band first appears near the band edge, consistent with state filling and the earliest buildup of population inversion. As the pump fluence is increased, the gain amplitude rises and the gain window broadens, extending progressively toward higher photon energies (λ < 600 nm). At the highest fluences, the bandwidth continues to expand rather than saturate, which is consistent with sequential population of higher lying excitonic transitions corresponding to 1P_e_1P_h_ states (containing up to 6 excitons per single emitting unit—likely a single facet). This suggests an efficient suppression of nonradiative Auger multicarrier decay even under strong excitation.

At the highest pump fluence, QCs exhibit an optical-gain lifetime of 0.34 ns, as shown in Figure 5d,f. We note that the presence of the long-lived PA signal in QCs (Figure S7c) shortens the time window of optical gain, which, however, is still comparable to best 2D and quasi-2D colloidal semiconductor samples, where gain lifetimes in the absence of semiconductor doping typically range from 30 to 600 ps.^77^ In particular, biexciton lifetimes, which usually exceed their corresponding gain lifetimes, span 60–400 ps in CdSe NPLs,^58,78^ 150–300 ps in CdS NPLs,^79^ and about 400–700 ps in optimally engineered CdSe/CdS isotropic core/shell and core/crown NPLs.^17,77^ It is also interesting to note that QCs exhibit a broader gain window of about 100 nm compared to less than 40 nm in the best core/crown NPL samples.^77^ This reflects the fundamentally different multiexciton interactions in the two systems: the multiexciton binding energy is negative in NPLs and positive in QCs, which helps mitigating nonradiative multicarrier losses in QCs.

Major progress has been made in reducing the mechanical fragility of standalone 2D nanosheets and NPLs, as well as in developing elegant assembly strategies that produce highly ordered stacks.^32,37^ For instance, ordered assemblies of 2D NPLs can be realized using tailored ligands or external electromagnetic fields.^9,80,81^ In this context, the QC geometry may offer further advances in achieving self-assembly of 2D colloids by enabling isotropic packing of 3D cubes into solid films that preserve the 2D excitonic behavior of CdSe facets.

To enable a more direct comparison between QC and NPL films under the same processing conditions, we avoided assembly specific optimization for either system. Accordingly, both 3-ML CdS/CdSe QC and 4-ML CdSe NPL films (with corresponding absorption profiles shown in Figure S9) were deposited by straightforward drop casting from equally concentrated solutions, minimizing the role of specialized assembly methods. As shown in Figures 6a and S10a–c, drop casting of CdS/CdSe QCs onto a TEM grid produces extended regions of ordered packing, indicating strong shape directed assembly under nonoptimized conditions. By comparison, conventional CdSe NPL films, prepared via drop casting, form less regular networks with larger anisotropic voids (Figures 6b and S10d–f).

**Figure 6:**
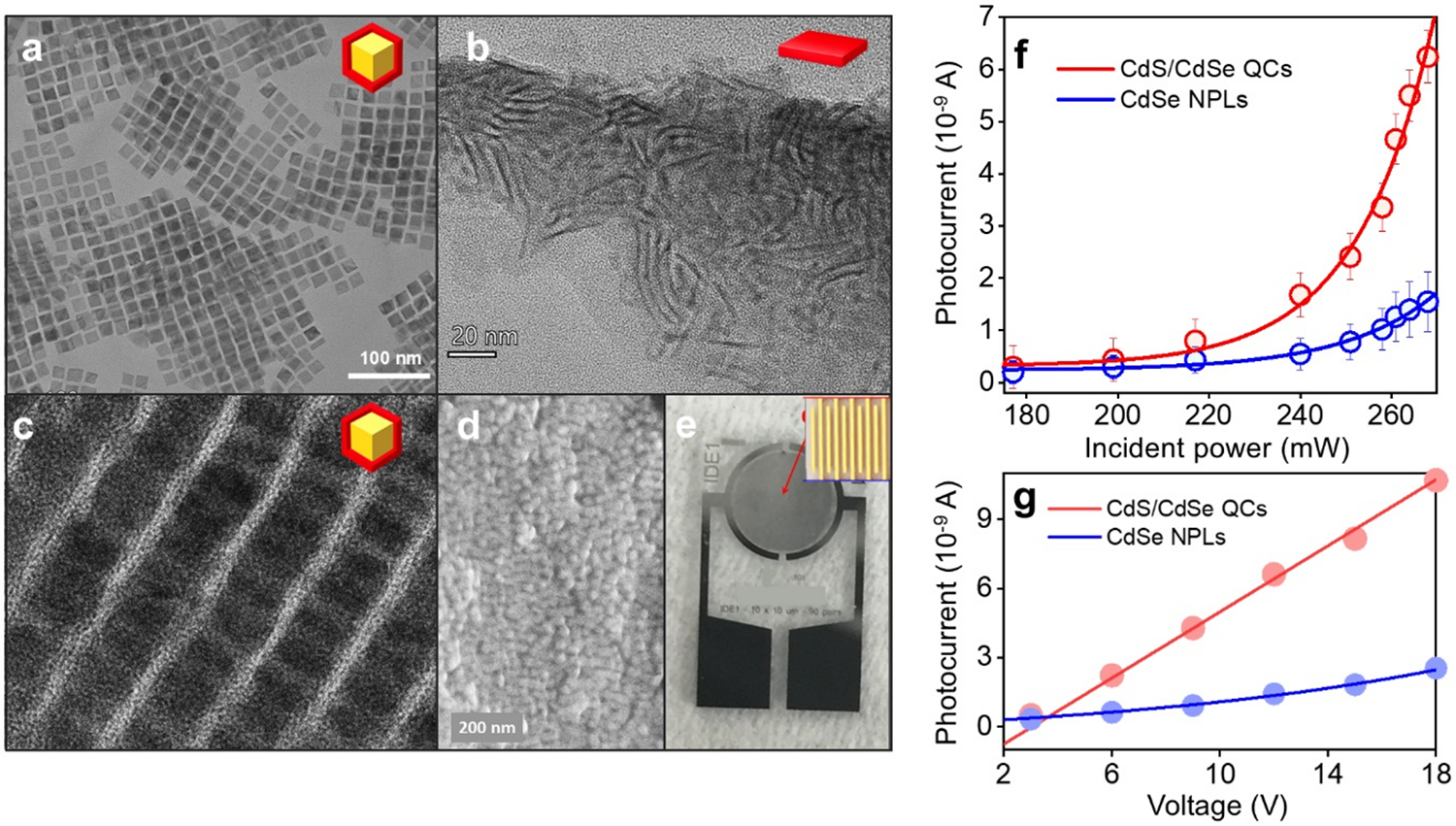
(a) TEM image of CdS/CdSe QCs drop cast on a TEM grid. (b) Representative drop cast film of conventional 2D NPLs. (c) Drop cast QC film prepared from a solution of self-assembled QCs. (d) SEM image of a QC film. (e) Schematic/optical image of the Micrux interdigitated electrode device used for photoconductivity measurements. (f) Photocurrent under simulated solar illumination for QC and NPL films with matched optical density, showing a typical ∼4.5× enhancement for CdS/CdSe QCs compared to CdSe NPLs at the same incident power. (g) Current–voltage characteristics of QC and NPL devices under illumination, exhibiting linear, Ohmic transport.

An enhanced regularity of solution-processed QC assemblies compared to NPLs suggests stronger interparticle coupling throughout the film, an important prerequisite for an efficient charge transport. Additionally, the surface localization of carriers in CdS/CdSe QCs is expected to further facilitate interparticle charge hopping, consistent with recent studies of inverted core/shell CdSe/PbSe QDs where surface-confined charges enhance electronic coupling.^82^ In order to directly compare electronic coupling of QCs and NPLs, both materials were drop casted on Micrux interdigitated electrode substrates (Figures 6e, and S11), followed by ligand exchange with short polar molecules to promote electrical coupling. For charge transport measurements, we employed ∼20 nm CdS/CdSe QCs with an approximate CdSe thickness of 3 MLs (Figure S10a) and without an outer CdS passivation shell to reduce tunneling barriers. Similarly, films of CdSe NPLs with an approximate thickness of 4 monolayers (Figure S10b) and lateral size of 20–40 nm were processed using very similar conditions.

As shown in Figure 6f, under simulated solar illumination, QC films exhibit a higher photocurrent, yielding on average a 4.5-fold enhancement relative to NPL films after adjusting for the absorbed energy (see SI section for further details). This trend was reproduced using several QC batches, confirming that the improvement arises from intrinsic differences in film morphology and coupling rather than sample-to-sample variation. We note that current–voltage (*I*–*V*) measurements for both QC and NPL films were close to linear over the applied bias range (Figure 6g), confirming Ohmic transport in both systems. This linearity rules out contact-limited or Schottky-barrier dominated conduction and indicates that the observed photocurrent enhancement in QC films originates from improved bulk transport. We attribute this behavior to the dense packing of QCs, which increases the number of electronic percolation pathways and reduces interparticle spacing relative to 2D NPL assemblies.

Improved photoconductivity in QC films versus NPLs can potentially benefit device performance across multiple platforms. In photodetectors, stronger electronic coupling improves charge extraction when interparticle transfer limits response. In electrically pumped lasers, higher film conductivity reduces resistive losses, lowering heating and reducing current thresholds. For photoanodes, QCs offer enhanced charge separation and reduced Auger recombination, helping mitigate recombination losses that constrain NPL based systems.^83^ Since higher photocurrent in 2D chalcogenides correlates with increased hydrogen evolution,^84^ QC films could support more efficient photoanodes for photoelectrochemical cell water-splitting and hydrogen evolution reaction (HER).

## Conclusions

In conclusion, we introduce colloidal semiconductor quantum cubes (QCs) as a supported-nanosheet architecture in which 2D CdSe quantum wells are grown on the six facets of bulk-like CdS nanocubes. This geometry combines desirable excitonic properties of quasi-2D semiconductors with the structural and electronic advantages of a rigid cubic scaffold. We show that the QC design gives rise to an unusual multiexciton regime, characterized by repulsive exciton–exciton interactions, which leads to suppressed Auger recombination, long multiexciton lifetimes, and broadband optical gain. Single-particle measurements further suggest that the six CdSe facets behave as weakly coupled, quasi-independent emitters. At the same time, the cubic shape promotes more ordered self-assembly on substrates and improves interparticle coupling in solution-processed films without extensive postsynthetic optimization. Overall, colloidal QCs provide a practical route toward electrically integrated, solution-processed photonic materials that combine structural robustness with quasi-2D excitonic functionality.

## Methods

### Materials

The following chemicals were used as received without further purification or modification: anhydrous acetone (99%, Amresco), cadmium oxide (CdO, 99.95%, MilliporeSigma), zinc acetate dihydrate (98%, Acros Organics), zinc chloride (ZnCl_2_, ≥98%, MilliporeSigma), anhydrous ethanol (EtOH, 99%, BeanTown Chemical), hexane (ACS grade, Thermo Scientific), 1-octadecene (ODE, technical grade, 90%, MilliporeSigma), octane (98%, MilliporeSigma), 1-octanethiol (97%, Alfa Aesar), oleic acid (OA, technical grade, 90%, MilliporeSigma), oleylamine (OLAM, technical grade, 70%, MilliporeSigma), dioctylamine (DOA, 97%, MilliporeSigma), rhodamine 101 inner salt (R101, 94%, Thermo Scientific), selenium powder (Se, 99.5%, 200 mesh, Thermo Scientific), sulfur powder (S, 99.999%, Thermo Scientific), toluene (99.8%, MilliporeSigma), and tri-n-octylphosphine (TOP, 97%, Strem Chemical). Tellurium powder (Te, 99.8%, MilliporeSigma), cadmium acetate (Cd­(OAc)_2_, ≥98%), and zinc acetate (Zn­(OAc)_2_, ≥ 98%) were also used as specified. High-purity argon was employed for inert-atmosphere operations.

### Synthesis of Bulk-Size CdS Cubes

Large size CdS cubes were synthesized by slight modification in the reported procedure.^43,85^ To this end cadmium acetate 0.092 g, decanoic acid 0.32 g, 1.5 and 9 mL of oleic acid and octadecene respectively were added to a 50 mL flask and kept under vacuum for 15 min at 150 °C. The mixture was then kept under an argon atmosphere by using a Schlenk line and heated until 270 °C. At 270 °C 0.4 mL stearoyl chloride was injected into the reaction flask. After that 146 nmols of small CdS seeds were injected and the reaction temperature was raised to 295 °C, resulting in the fusion of smaller CdS NCs into larger cube. As an optional step to achieve an accelerated fusion, 0.03 M S-ODE precursor was injected at the rate of 0.7 mL/h for 4h at 295 °C. The final product was then purified by adding equal volume of ethanol to the nanoparticle solution and centrifuging at 7500 rpm for 5 min. The supernatant was discarded, and the precipitate was dispersed in toluene.

### Synthesis of CdS/CdSe Core/Shell Cubes

To grow a cubic CdSe shell over large size CdS cubes cadmium acetate 0.092 g, decanoic acid 0.12 g, erucic acid 0.25 g and 9 mL octadecene respectively were added to a 50 mL flask and kept under vacuum for 15 min at 150 °C. The mixture was then kept under an argon atmosphere by using a Schlenk line and heated until 270 °C. At 270 °C 0.7 mL stearoyl chloride was injected into the reaction flask. After that 147 nmols of bulk size CdS cubes (dispersed in ODE) were injected and the reaction temperature was raised to 295 °C. At 295 °C 0.03 M Se-ODE precursor was injected at the rate of 1 mL/h for 3h. The final product was then purified by adding 10 mL chloroform and 5 mL methanol to the nanoparticle crude solution in the 50 mL centrifuging tubes and centrifuged at 7500 rpm for 5 min. The supernatant was discarded, and the precipitate was dispersed in toluene.

### Synthesis of CdS/CdSe/CdS Core/Shell/Shell Cubes (CdSe QCs)

To synthesize CdSe QCs cadmium acetate 0.092 g, decanoic acid 0.12 g, erucic acid 0.25 g and 9 mL octadecene respectively were added to a 50 mL flask and kept under vacuum for 15 min at 150 °C. The mixture was then kept under an argon atmosphere by using a Schlenk line and heated until 270 °C. At 270 °C 0.7 mL stearoyl chloride was injected into the reaction flask. Then all of the synthesized CdS/CdSe core/shell QCs (dispersed in ODE) were injected and the reaction temperature was raised to 295 °C. At 295 °C 0.05 M S-ODE precursor was injected at the rate of 0.5 mL/h for 4h. The final product was then purified by adding 10 mL chloroform and 5 mL methanol to the nanoparticle crude solution in the 50 mL centrifuging tubes and centrifuged at 7500 rpm for 5 min. The supernatant was discarded, and the precipitate was dispersed in toluene.

### Synthesis of CdSe Nanoplatelets

CdSe NPLs (4 ML thickness) were synthesized with minor adjustments to the previously reported procedure.^86^ Cadmium oxide 75 mg, myristic acid 250 mg and octadecene 7 mL were added to a 50 mL flask and kept under vacuum for 15 min at 150 °C. The mixture was then kept under an argon atmosphere by using a Schlenk line and heated until 250 °C. Once the solution was clear 10 mL octadecene was injected and then 26 mg Selenium was added around 130 °C and temperature was raised to 215 °C. At 185 °C 125 mg cadmium acetate was added to the reaction mixture. Reaction was kept for 5 min at 215 °C and cooled down using water bath, while cooling down 4 mL of oleic acid was injected into the reaction flask. The final product was then purified by adding equal volume of ethanol to the nanoparticle solution and centrifuging at 7500 rpm for 5 min. The supernatant was discarded, and the precipitate was dispersed in toluene.

### Oleic Acid Treatment of CdS/CdSe QCs

Synthesized CdS/CdSe QCs were loaded in the flask with a 75:25 mixture of OA: ODE and the reaction was kept at 130 °C for 30 min. Same procedure was repeated with CdSe NPLs.

### Sample Characterization

Absorption spectra were recorded using a Cary 60 UV–vis spectrophotometer. Photoluminescence spectra were measured under excitation from a pulsed 405 nm laser diode (PDL 800-D, PicoQuant), with the emitted light collected through a fiber-optic cable and analyzed using an Andor Shamrock 303i spectrograph coupled to an Andor Newton 970 EMCCD detector. TEM images were acquired on a Thermo Fisher Talos F200X G2 S/TEM operated at 200 kV using samples deposited on carbon-coated 300-mesh copper grids. Time-resolved emission lifetime spectra were recorded using the same 405 nm pulsed laser, and photons were collected and processed using a SPC-130 TCSPC module from Becker & Hickl. Absolute PL quantum yields were measured with a Quantaurus-QY C11347 absolute PL quantum yield spectrometer (Hamamatsu, Japan).

### Transient Absorption Measurements

Samples were excited with 170 fs pump pulses at 515 nm, generated by second-harmonic conversion in an α-BBO crystal. Probe pulses were produced from the 1030 nm fundamental in a 2 cm YAG crystal. The temporal delay between pump and probe was controlled with a delay stage, providing delays of up to 6 ns for 515 nm excitation. In our measurements, the probe spectrum covered the 515–900 nm range. The QCs were dispersed in optically transparent hexane and continuously stirred during the experiment to minimize charging and photodegradation. The pump wavelength and sample concentration were chosen to provide a good signal at the band-edge transitions while avoiding excessive absorption at the pump wavelength, thereby maintaining more uniform excitation throughout the sample.

### Single-Particle Spectroscopy

A concentrated solution of QCs in hexane was diluted and dispersed onto a glass substrate to achieve an approximate surface density below 0.01 particles per μm^2^. The sample was mounted on the translation stage of an optical microscope and excited with 405 nm, 50 ps laser pulses through a 100×, 1.2 NA oil-immersion objective, which was also used to collect the PL signal. The laser repetition rate was adjusted to ∼1–2 MHz so that the interval between pulses was much longer than the PL decay time, ensuring complete exciton relaxation between successive excitation events. The emitted PL was directed to two PerkinElmer avalanche photodiodes (SPCM AQR-13) arranged in a standard Hanbury Brown-Twiss geometry using a 50/50 beamsplitter. Time-tagged, time-correlated single-photon counting (TCSPC) measurements were performed using PicoQuant MultiHarp 150 electronics. This setup records photon arrival times both relative to the start of the measurement cycle and relative to the excitation pulse, allowing PL decay curves to be reconstructed for selected time intervals of the PL intensity trajectory or for chosen regions of the intensity distribution. Homemade IGOR software was used to process the photon streams from both detection channels to generate time-gated *g*(2).

### Film Preparation for Photoconductivity Measurements

Micrux thin-film interdigitated electrodes were sonicated in isopropyl alcohol and dried. A few drops of oleic-acid-treated CdS/CdSe cubes were then drop-cast onto the electrodes. To perform ligand exchange, a few drops of ammonium thiocyanate solution were drop-cast onto the film, allowed to react for 1–2 min, and then rinsed with several drops of methanol. This treatment replaces oleic acid with short, polar ligands, improving electrical coupling between neighboring nanoparticles. The same procedure was used to prepare films of CdSe NPLs. The drop-cast concentrations of cubes and NPLs were kept approximately the same for all samples.

## Supplementary Material



## Notes

Spectroscopy characterization of QCs, additional TEM images, rate equations and simulations; Methods Section (PDF)
