# Supplementary material for: 2D Semiconductor Nanosheets Supported on Colloidal Quantum Cubes
Source: ACS Nano. 2026 Jun 22;20(26):18728–39. doi: 10.1021/acsnano.6c03530 (PMC13348164; doi:10.1021/acsnano.6c03530)
Supplement: Supplementary file 1 [file nn6c03530_si_001.pdf]

Supporting Information for:

2D Semiconductor Nanosheets Supported on Colloidal Quantum Cubes

Divesh Nazar[1,2], Dulanjan Harankahage[1,2], Annelies Vitharana[3,4], Benjamin T. Diroll[5], Mykhailo V. Bondarchuk[1,6], Christopher M. Hicks[1,6], Sean Smith [7], Dmitry Porotnikov,[1,2] Amelia D. Waters[1,2], Siddhartha Thennakoon[1,2], Kosgoda Somarathne[1,2], Joelle DesAutels,[2] Issatay Nadinov,[8] Krishna P. Acharya,[9] Richard D. Schaller,[5,10] Alexander N. Tarnovsky[1,6], Anton V. Malko[7], Pieter Geiregat[3,4], Mikhail Zamkov[1,2,].*

The Center for Photochemical Sciences, Bowling Green State University, Bowling Green, Ohio 43403.[1]

Department of Physics, Bowling Green State University, Bowling Green, Ohio 43403.[2]

Physics and Chemistry of Nanostructures, Ghent University, 9000 Gent, Belgium[3]

NOLIMITS Center for Non-Linear Microscopy and Spectroscopy, Ghent University, 9000 Gent, Belgium[4]

Center for Nanomaterials, Argonne National Laboratory, Lemont, IL, 60439, USA[5]

Department of Chemistry, Bowling Green State University, Bowling Green, Ohio 43403.[6]

Department of Physics, The University of Texas at Dallas, Richardson, Texas, 75080[7]

Center for Renewable Energy and Storage Technologies (CREST), Division of Physical Sciences and Engineering, King Abdullah University of Science and Technology (KAUST), Thuwal, Kingdom of Saudi Arabia.[8]

Savannah River National Laboratory, Aiken, SC 29808, USA[9]

Department of Chemistry, Northwestern University, Evanston, Illinois 60208.[10]

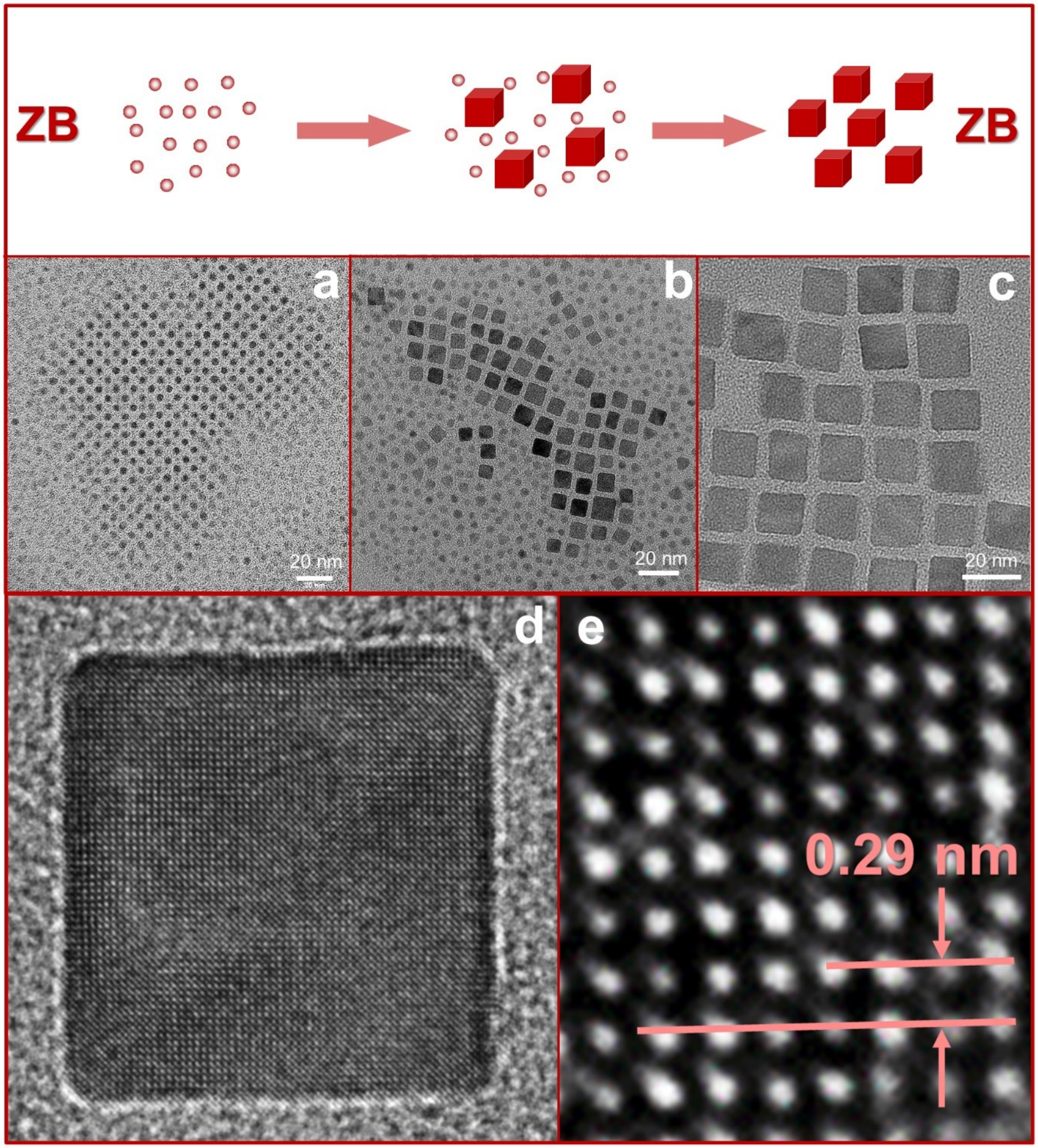

Figure S1. (a-c). Evolution of the CdS NC shapes during aggregative growth in OA/$C_{17}H_{35}COCl$. At an early stage in (a), two populations of NCs are observed, including original NC seeds and cube-shaped assemblies. (b,c) After ~ 2–3 h at 300 °C, the initial NC seeds are largely depleted and cube-shaped NCs become the dominant product. The resulting cubes adopt the zinc blende (ZB) crystal structure. (d) High-resolution TEM image of a representative CdS cubic core, showing a single-crystal lattice formed through the coalescence of smaller CdS NCs. (e) The corresponding lattice spacing is consistent with ZB CdSe.

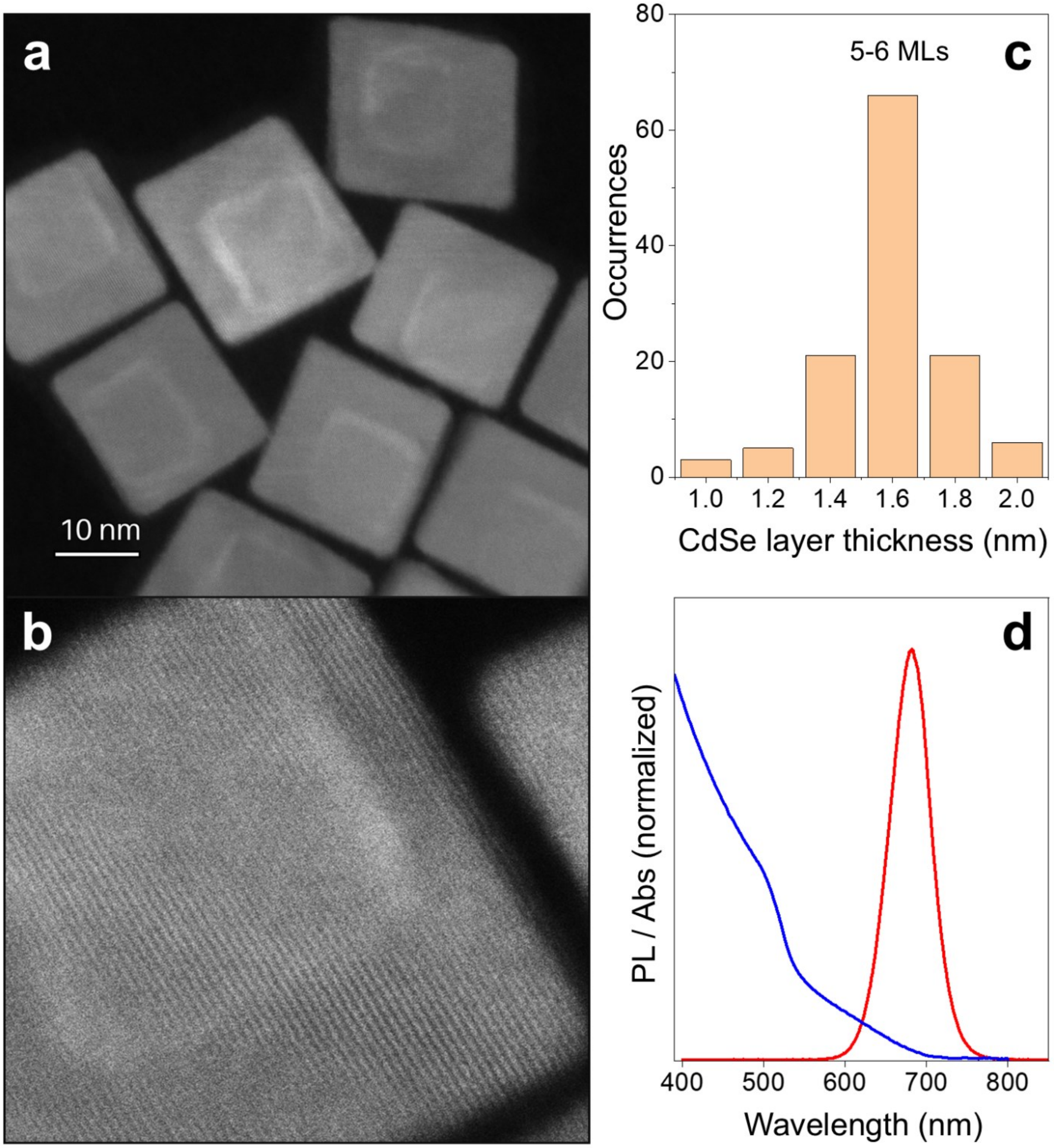

Figure S2. (a,b) High-resolution HAADF-STEM images of CdS/CdSe/CdS quantum cubes (QCs), in which the CdSe 2D nanosheets appear with lighter contrast relative to the CdS core. (c) Statistical analysis of the CdSe layer thickness measured from the structures, shown in panel a, yielding an average thickness of 1.6 nm. (d) Absorption and emission spectra of the QCs shown in panels a and b.

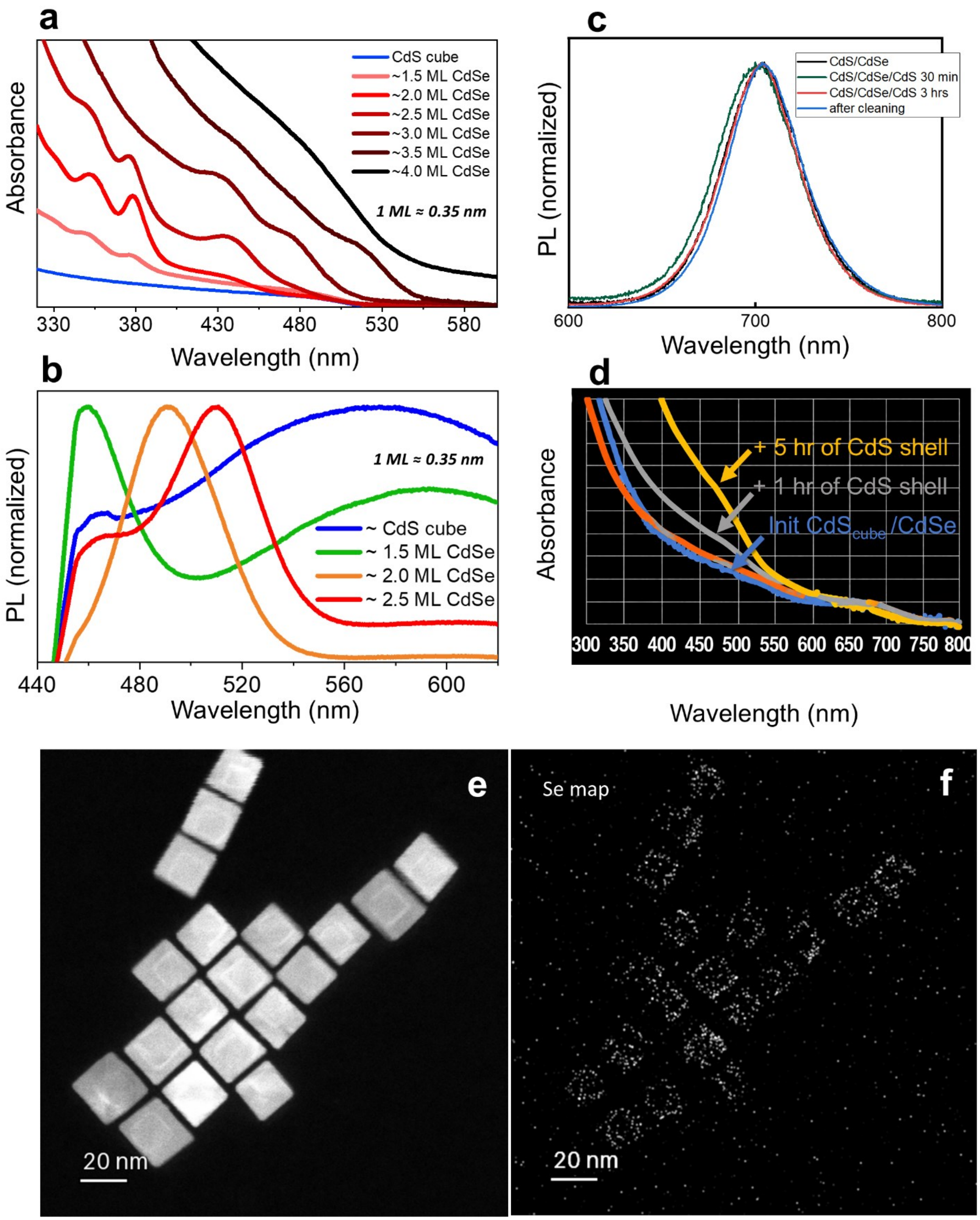

Figure S3. (a) Evolution of the absorption spectra during CdSe shell growth on CdS/CdSe quantum cubes, with the approximate CdSe thickness indicated in monolayers (1 ML = 0.35 nm).

(b) Evolution of the PL spectra for samples with different CdSe thicknesses. (c) Evolution of the PL spectra following growth of the outer CdS shell on CdS/CdSe core cubes. (d) Evolution of the absorption spectra following growth of the outer CdS shell on CdS/CdSe core cubes. (e) HAADF-STEM image of CdS/CdSe/CdS QCs, along with EDAX elemental mapping of Se demonstrating about 1:3 ratio of facet-to-edge Se.

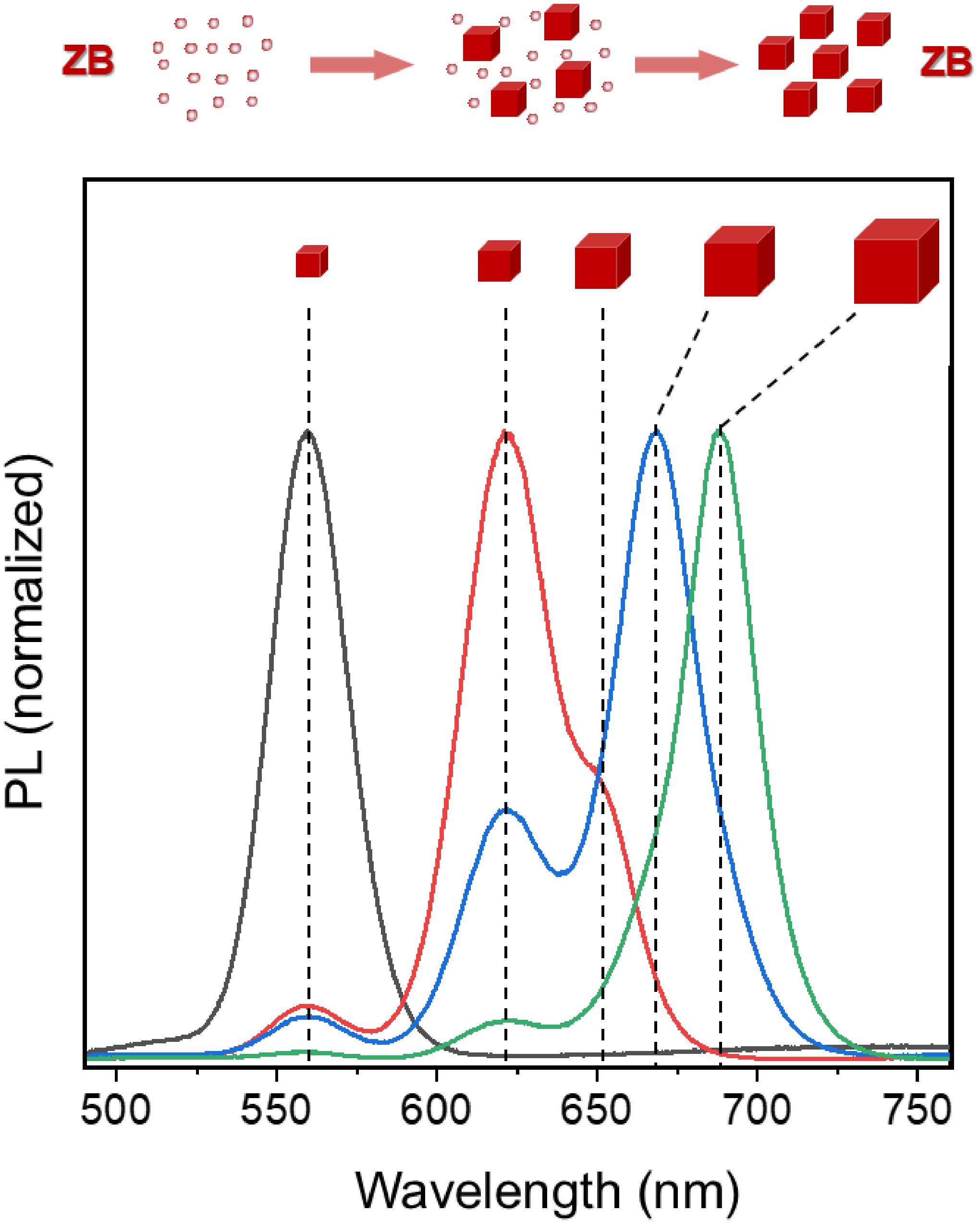

Figure S4. Mechanism of cadmium chalcogenide cube growth revealed using an example of CdSe. Evolution of CdSe NC shapes during aggregative growth in OA/$C_{17}H_{35}COCl$, recorded starting from the room temperature. The pattern reveals multi-modal size distributions with blue-shifted peak arising from smaller particles and red-shifted features from fused 2, 3, … - particle products.

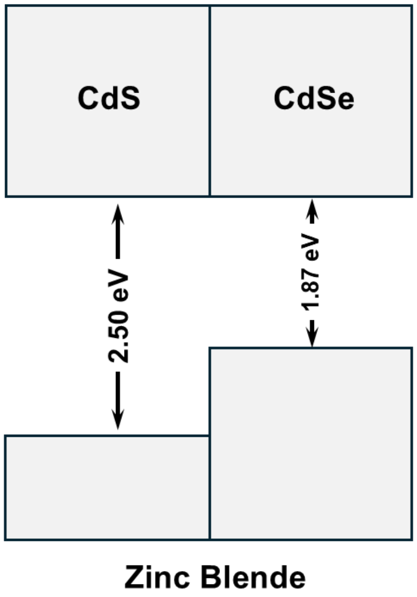

Figure S5. Band edge alignment of zinc blende CdS and CdSe (bulk) semiconductors.

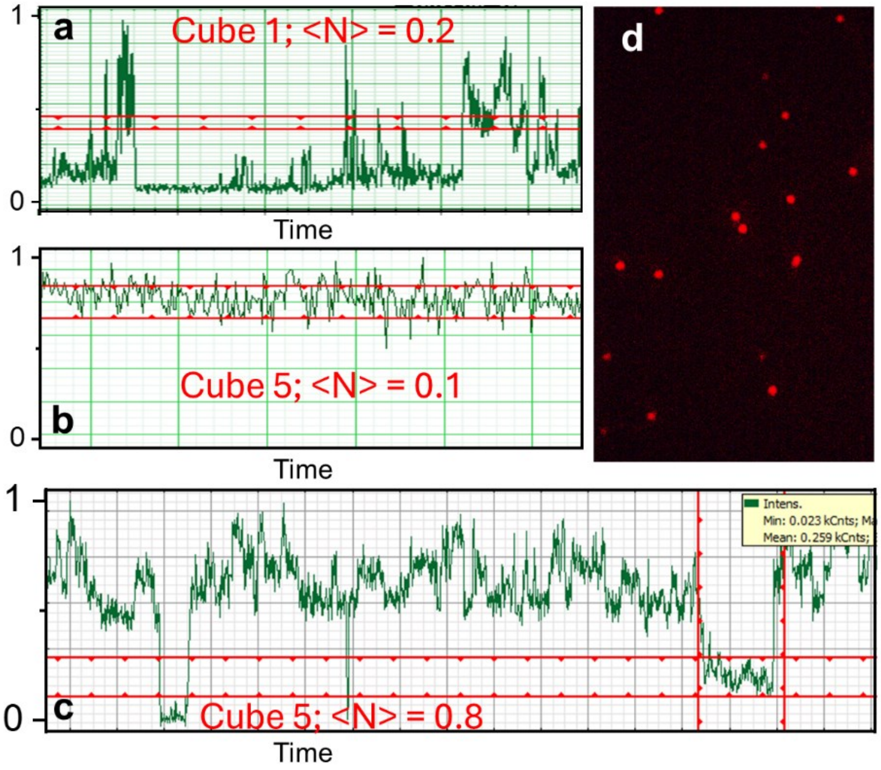

Figure S6. Single-particle PL trajectories of individual QCs under different excitation conditions. (a) PL trajectory of a single QC 1 showing pronounced blinking at low excitation fluence (⟨N⟩ = 0.2). (b) PL trajectory of QC 5 showing strongly suppressed blinking at low excitation fluence (⟨N⟩ = 0.1).

(c) PL trajectory of QC 5 at higher excitation fluence ($\langle N \rangle = 0.8$), exhibiting grey-state emission. (d) a representative image of individual QCs used in single-particle measurements.

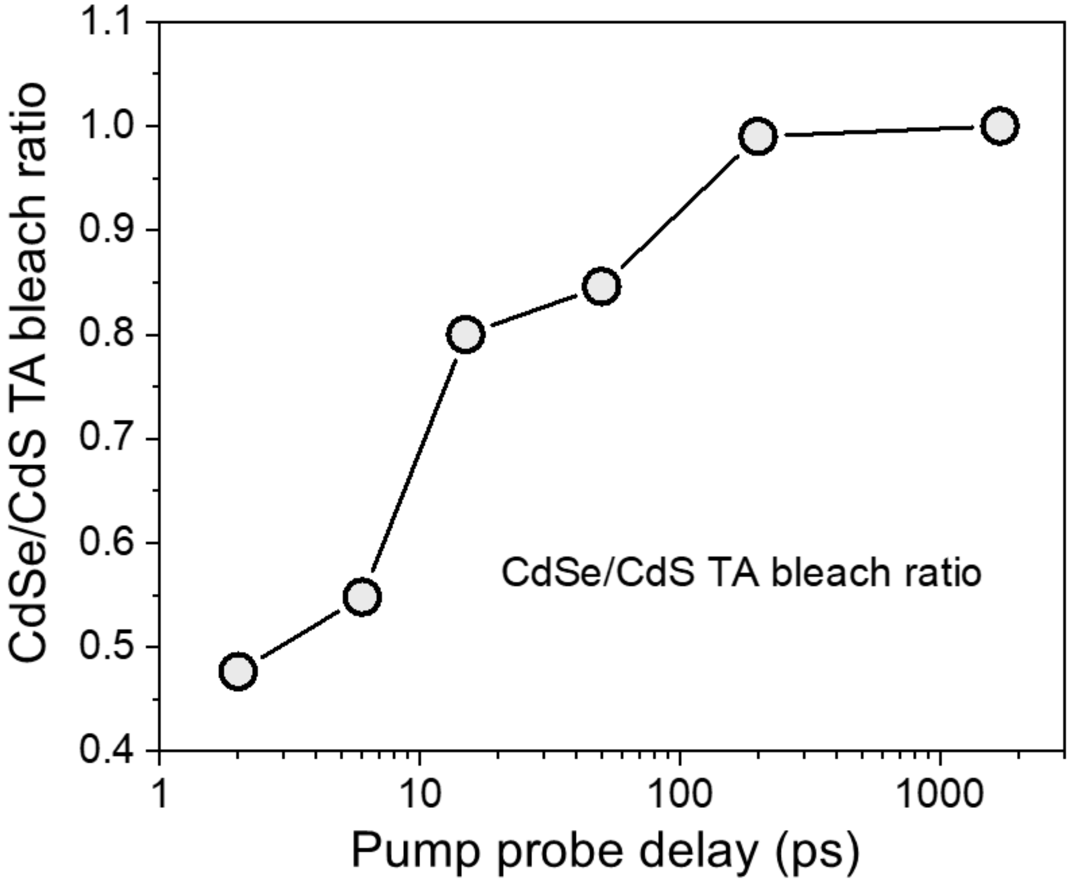

Figure S7. Transient-absorption bleach amplitude ratio between the CdSe- and CdS-associated features in quantum cubes. The increasing CdSe-to-CdS bleach fraction indicates time-dependent carrier redistribution, consistent with charge migration from CdS regions into the CdSe nanosheet domains.

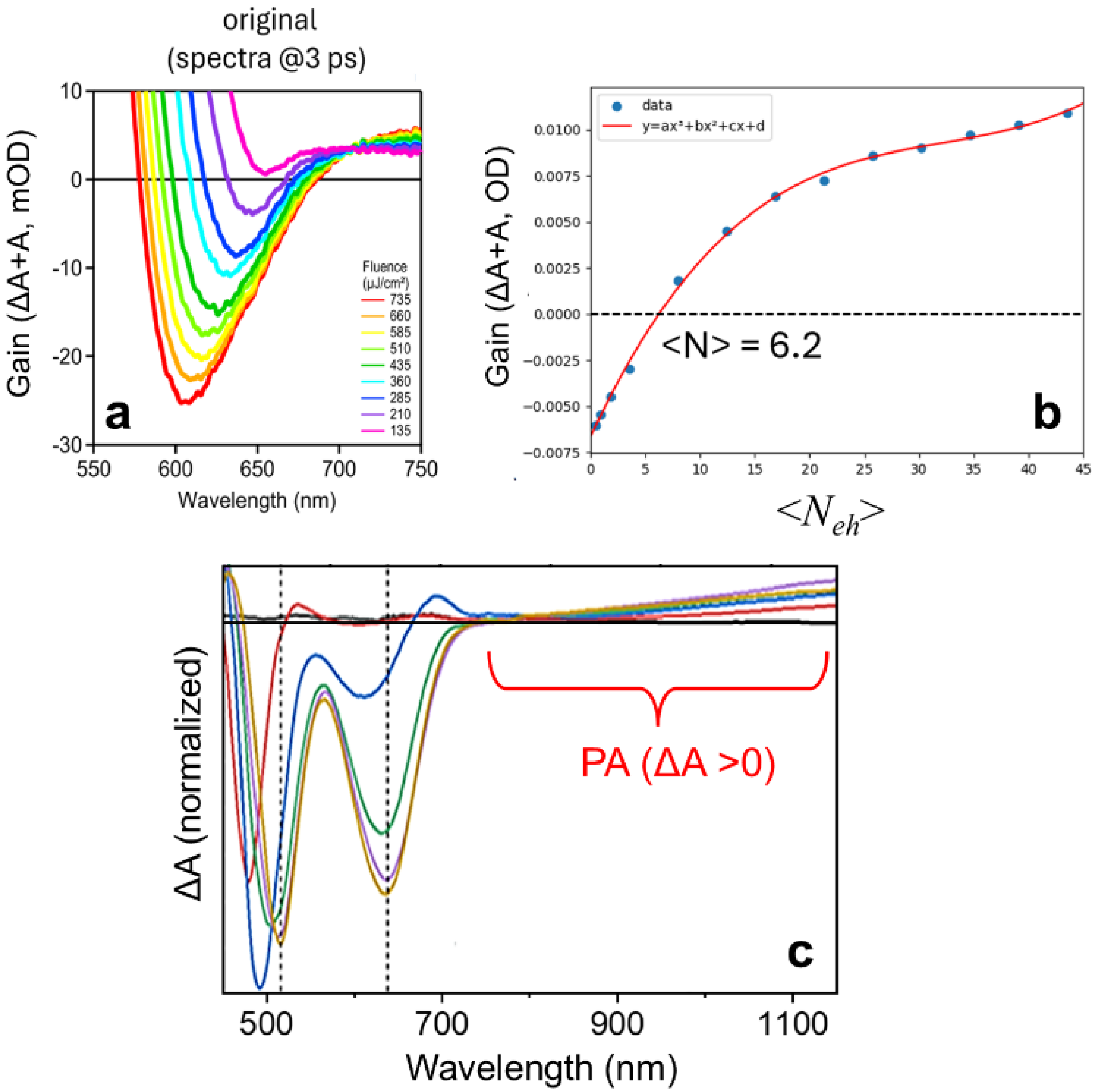

Figure S8. (a) The gain spectra corresponding to the average occupation, $\langle N_{eh} \rangle$, prior to subtracting the PA contribution. (b) Gain at λ = 660 nm versus $\langle N_{eh} \rangle$. (c). Extending the spectral window of the TA spectra into near-IR demonstrates a long-lived PA, which competes with optical gain all the way down to the 650-750 nm range.

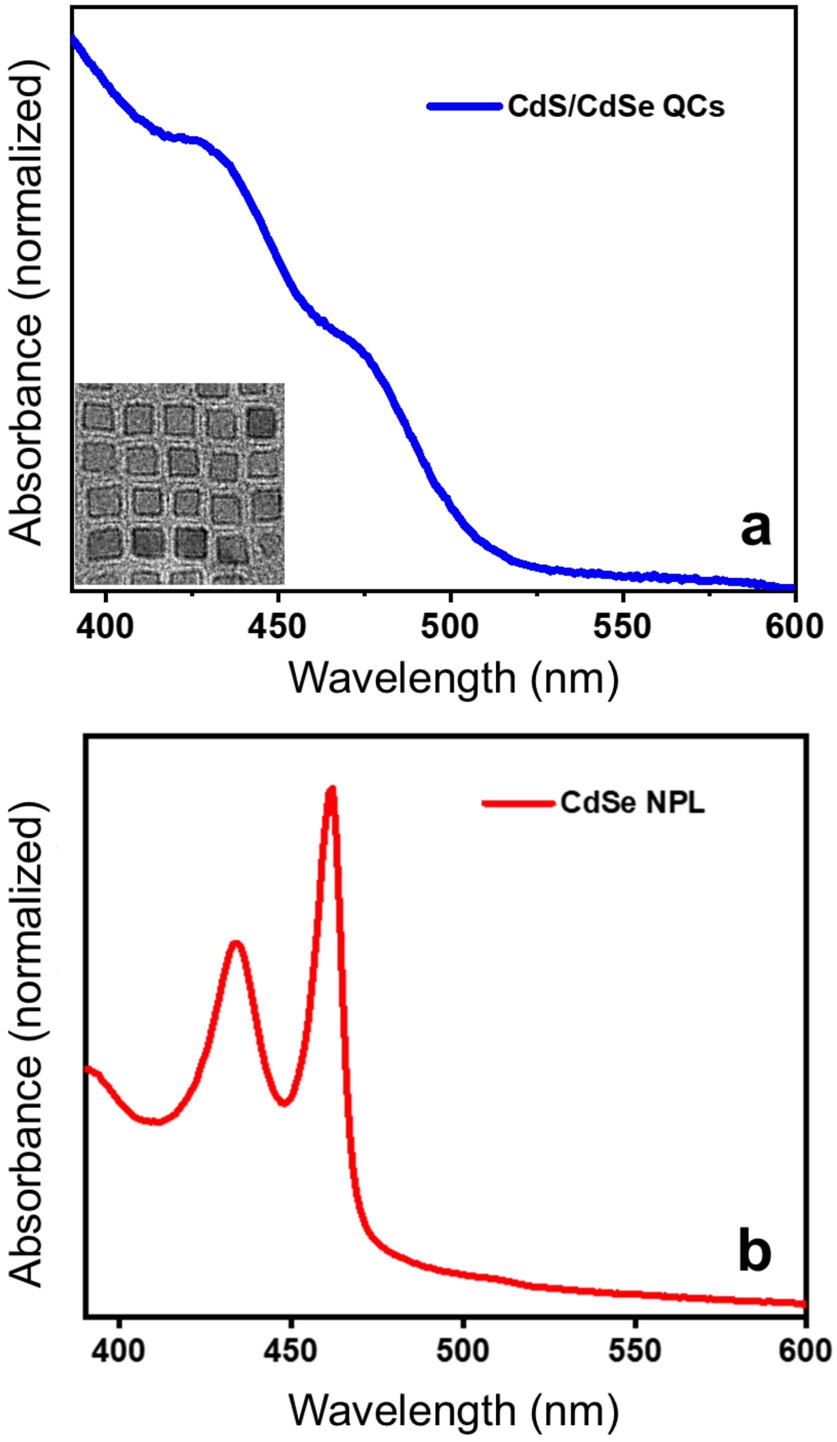

Figure S9. Characterization of nanostructures used in photoconductivity measurements. (a) Absorption spectrum of CdS/CdSe QCs with an approximate CdSe thickness of 3 MLs. (b) Absorption spectrum of CdSe nanoplatelets with an approximate thickness of 4 MLs.

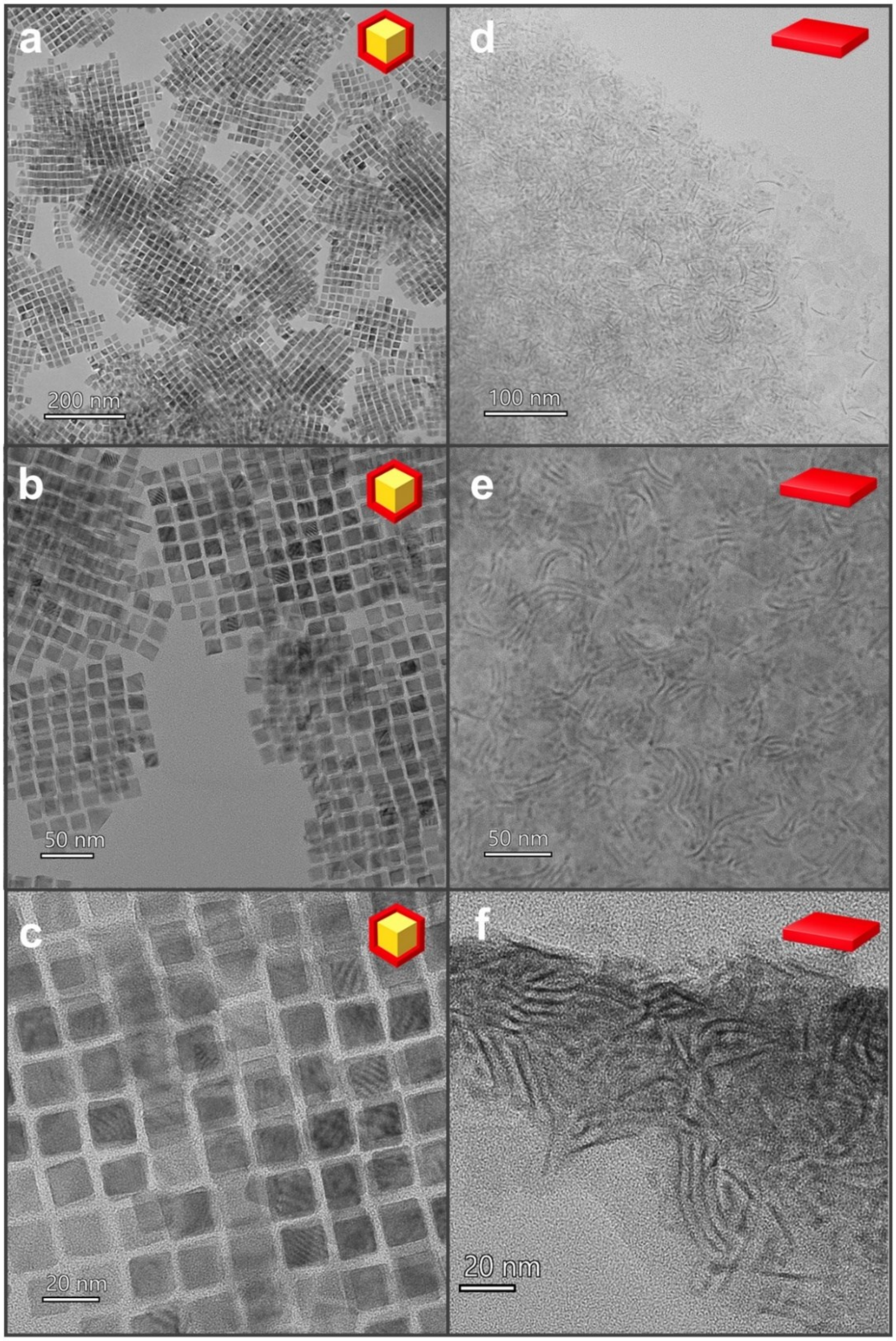

Figure S10. TEM images of drop-cast NC assemblies on TEM grids. (a-c) – quantum cubes. (d-f) – CdSe nanoplatelets.

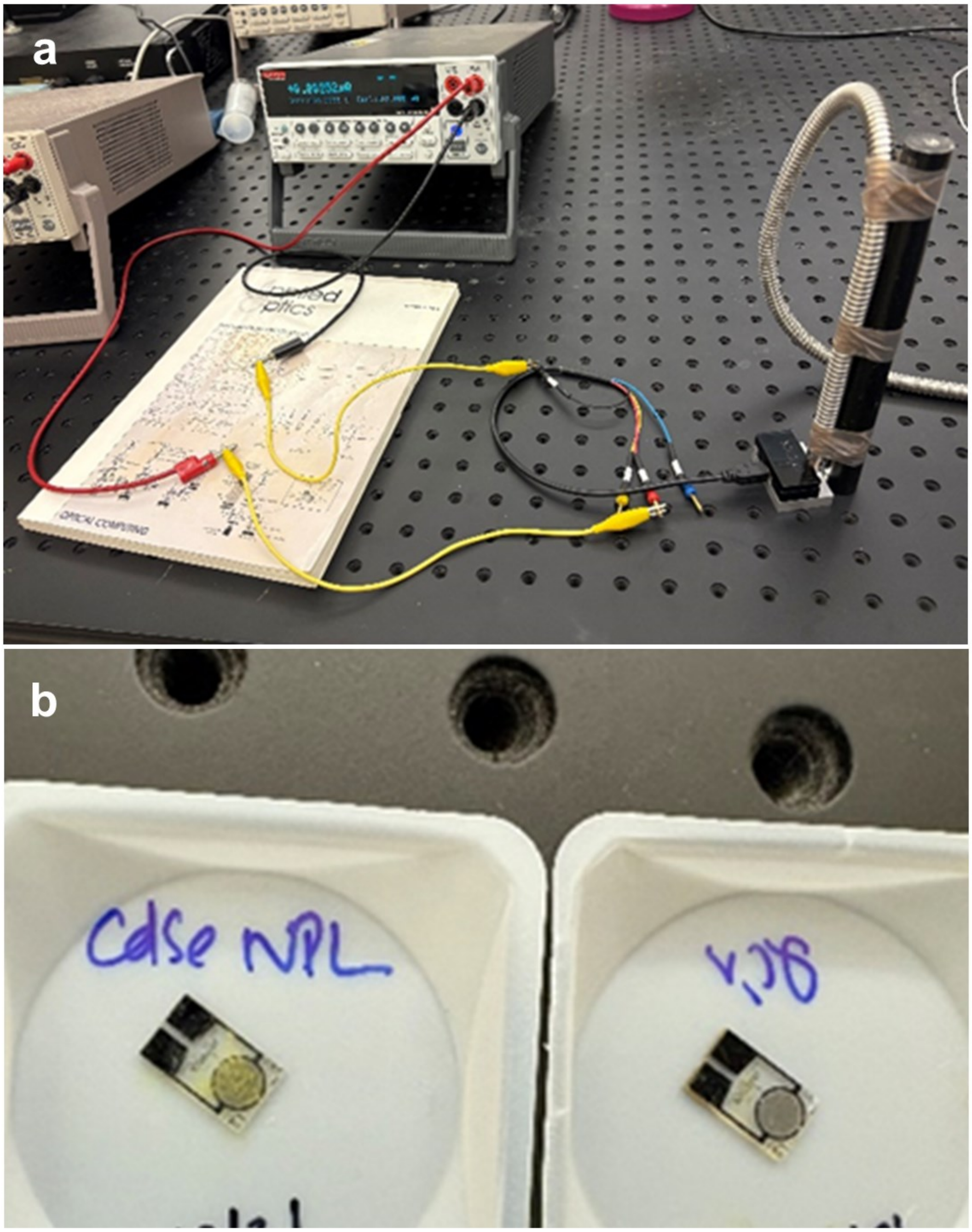

Figure S11. Experimental setup for measuring the photoconductivity of colloidal NC assemblies. (a) Image of the sample, illuminated using optical fiber and read out by the USB port, interfaced with Keithley. (b) Images of nanoplatelets (left) and QCs (right) on top of Micrux substrates.

Section I. PL intensity decay fitting procedure:

The experimental PL intensity decay in Figure 3b of the main text was compared to the model calculations of PL emission from a multi-exciton state. The model estimated the time evolution of PL for a given average number of excitation photons per particle, $\langle N_{eh} \rangle$, with biexciton emission quantum yield, QY_{XX}, being the only fitting parameter. The value of $\langle N_{eh} \rangle$ was first used to determine the initial multiexciton population distribution using the Poisson formula:

$$f(m) = \langle N_{eh} \rangle^{m} \times e^{-m} / m! \quad \text{(SE1)}$$

where m is the number of excitons per particle.

For each excitonic state m, we can then determine the radiative ($k_{m,r}$) and nonradiative ($k_{m,nr}$) decay rates using statistical scaling of exciton decay rate by Klimov et al., [1]:

$$k_{m,r} = \mathrm{m}^2 k_{r,2}/4, \qquad k_{m,nr} = \mathrm{m}^2(\mathrm{m}-1)\, k_{nr,2}/4 \quad \text{(SE2)}$$

where $k_{r,2}$ and $k_{nr,2}$ are the corresponding radiative and nonradiative rates for m = 2 populations (biexcitons). In this work, these rates were determined using a fitting parameter, QY_{xx}, and known values of a single-exciton total decay constant, $\tau_{tot,1}$, and the single-exciton state PL QY, QY_X:

$$k_{2,r} = 2^2\, \mathrm{k}_{r,1} = 4 \times \mathrm{QY_X}/\tau_{tot,1} \quad \text{(SE3)}$$

$$k_{2,nr} = 4 \times \frac{1-QY_{xx}}{QY_{xx}} \times k_{1,r} = 4 \times \frac{1-QY_{xx}}{QY_{xx}} \times \frac{QY_x}{\tau_{tot,1}} \quad \text{(SE4)}$$

Time-dependent population dynamics were then computed using a finite-difference time-step approach, accounting for radiative decay, nonradiative decay, and cascading transitions between adjacent exciton states. Considering that Auger decay of a m-exciton state results in a state with (m - 1) excitons, the temporal evolution of the m-exciton population in a QS, $P(m, t)$, is then determined by solving coupled rate equations:

$$\frac{dP(m,t)}{dt} = k_{m+1} P(m+1,\, t) - k_m P(m,\, t) \quad \text{(SE5)}$$

where, $k_m = k_{m,r} + k_{m,nr}$, represents the total decay rate of an m-exciton state.

Section II. Determination of absorption cross-section from the TEM images for QCs:

The absorption cross-section, σ of QCs was determined using the formula:

$$\sigma_\omega = \frac{4\pi\alpha\omega}{n_{medium}} |f(\omega)|^2 R^3$$

where:

- σ_ω = absorption cross section at angular frequency ω
- α = fine structure constant ($\frac{e^2}{\hbar c} \approx \frac{1}{137}$)
- ω = angular frequency of the incident light (in rad/s)
- $f(\omega)$ = oscillator strength of the electronic transition at frequency ω
- n_{medium} = refractive index of the surrounding medium
- R = radius of the nanocrystal

This expression can be simplified as:

$$\sigma_\omega = \xi(\omega) R^3$$

where $\xi(\omega)$ is a material and transition-specific constant that incorporates the optical matrix elements and environmental parameters. For instance, in the case of CdS/CdSe/CdS QCs with the total edge length of a, the cross section was calculated as:[2]

$$\sigma_{3.1eV} = 6.55 \times 10^{-16} \times (a/2)^3 (nm)[cm^2] \quad \text{(SE8)}$$

References.

[1] Klimov, V. I.; McGuire, J. A.; Schaller, R. D.; Rupasov, V. I. Scaling of Multiexciton Lifetimes in Semiconductor Nanocrystals. *Phys. Rev. B* **2008**, *77* (19), 195324.

[2] Guzelturk, B.; Diroll, B. T.; Cassidy, J. P.; Harankahage, D.; Hua, M.; Lin, X.-M.; Iyer, V.; Schaller, R.D.; Lawrie, B.; Zamkov, M. Bright, Fast, and Durable Scintillation from Colloidal Quantum Shells. *Nature Comm.* **2024**, *15*, 4274.
